# Quantitative Proteomic Analysis of the Response to Cold Stress in Jojoba, a Tropical Woody Crop

**DOI:** 10.3390/ijms20020243

**Published:** 2019-01-09

**Authors:** Fei Gao, Pengju Ma, Yingxin Wu, Yijun Zhou, Genfa Zhang

**Affiliations:** 1 College of Life and Environmental Sciences, Minzu University of China, Beijing 100081, China; s161088@muc.edu.cn (P.M.); 16051039@muc.edu.cn (Y.W.); zhouyijun@muc.edu.cn (Y.Z.); 2 Beijing Key Laboratory of Gene Resource and Molecular Development, Beijing 100875, China; 3 College of Life Sciences, Beijing Normal University, Beijing 100875, China

**Keywords:** *Simmondsia chinensis*, cold stress, proteomics, leaf, iTRAQ

## Abstract

Jojoba (*Simmondsia chinensis*) is a semi-arid, oil-producing industrial crop that have been widely cultivated in tropical arid region. Low temperature is one of the major environmental stress that impair jojoba’s growth, development and yield and limit introduction of jojoba in the vast temperate arid areas. To get insight into the molecular mechanisms of the cold stress response of jojoba, a combined physiological and quantitative proteomic analysis was conducted. Under cold stress, the photosynthesis was repressed, the level of malondialdehyde (MDA), relative electrolyte leakage (REL), soluble sugars, superoxide dismutase (SOD) and phenylalanine ammonia-lyase (PAL) were increased in jojoba leaves. Of the 2821 proteins whose abundance were determined, a total of 109 differentially accumulated proteins (DAPs) were found and quantitative real time PCR (qRT-PCR) analysis of the coding genes for 7 randomly selected DAPs were performed for validation. The identified DAPs were involved in various physiological processes. Functional classification analysis revealed that photosynthesis, adjustment of cytoskeleton and cell wall, lipid metabolism and transport, reactive oxygen species (ROS) scavenging and carbohydrate metabolism were closely associated with the cold stress response. Some cold-induced proteins, such as cold-regulated 47 (COR47), staurosporin and temperature sensitive 3-like a (STT3a), phytyl ester synthase 1 (PES1) and copper/zinc superoxide dismutase 1, might play important roles in cold acclimation in jojoba seedlings. Our work provided important data to understand the plant response to the cold stress in tropical woody crops.

## 1. Introduction

Jojoba, *Simmondsia chinensis* (link) Schneider, also called wild hazel, deer nut, oat nut and coffeeberry, is an important and unique oil crop. The importance lies in the crushing oil of its seeds—jojoba oil has a wide range of commercial applications, including cosmetic formulations, food products and aerospace lubricants [1]. The composition and physical properties of the oil extracted from Jojoba seeds are similar to those of sperm oil and thus jojoba oil is a promising alternative to the threatened sperm whale oil [2]. Its uniqueness lies in two aspects, on the one hand, jojoba oil is a kind of vegetable oils with unique physical property and no other vegetable oil has physical properties comparable to jojoba oil. On the other hand, jojoba is a dryland crop and jojoba can be grown in deserts and various arid land areas without competing with common crops for farmland. Jojoba exhibit extremely high level of tolerance to drought and high temperature stresses and jojoba is proposed to have the ability to curb desert expansion around the world [3].

Jojoba is a desert shrub native to the semi-arid region of the Sonoran desert at the junction of Mexico and USA. Since the discovery of the fine properties of jojoba, has been successfully introduced into tropical and subtropical regions of many other countries, such as Australia, India, Egypt and China [4]. Although Jojoba has high tolerance to drought and high temperature, it is sensitive to cold stress. Hindered by the low tolerance to low temperature stress, jojoba is difficult to grow in temperate zones. Especially, although jojoba has been successfully introduced in parts of Yunnan and Sichuan province, China, many introduction studies in temperate regions of China like Henan province have failed [5]. It is necessary to analyze the physiological and biochemical response of jojoba to the cold stress and to investigate the response of jojoba to cold stress at the molecular level.

Low temperature is one of the key environmental cues that negatively affect plant growth and development and limit the geographic distribution area of plants. To understand the plant response to low temperature stress, researchers have conducted a number of physiological, biochemical and molecular biological studies [6]. Through these results, we learned that, upon perception of the low temperature signal in plants, the stress signal is transmitted downstream to activate many transcription factors mediating stress tolerance and modulate the expression levels of many cold-responsive genes, finally leading to adjustment of a large number of biological processes, including photosynthesis, signaling, transcription, metabolism, cell wall modification and stress response [7]. However, most of the studies on plant responses to cold stress were conducted in model plants and common crops such as Arabidopsis [8], rice [9] and wheat [10], no systematic analysis of the cold stress response in jojoba was reported by far, despite its importance as a unique semi-arid, oil-producing industrial crop.

Since proteins are the key players in the majority of cellular biological processes, proteomics techniques have been the powerful tools for detection of the quantitative alterations in protein abundance in plant response to environmental stress. The classical proteomics approach was two-dimensional gel electrophoresis (2-DE) coupled with mass spectrometry (MS) identification. With the rapid development of quantitative MS, the gel-based proteomic techniques are gradually giving way to some newly-developed technologies, for example, stable isotope labeled quantitative proteomics methods such as the isobaric tags for relative and absolute quantitation (iTRAQ) labeling technique. iTRAQ coupled to liquid chromatography-quadrupole mass spectrometry (LC-MS/MS) represents an efficient proteomic approach for the fast identification and accurate quantification of the high complexity protein mixture [11] and is currently being widely used for the quantitative comparative analysis of plant proteomes to various environmental stresses [12,13,14,15].

In the present study, the physiological and proteomic responses of jojoba to cold stress were investigated using iTRAQ-coupled LC-MS/MS technique. This study will reveal how leaf proteins and their related pathways were regulated for jojoba’s response to cold stress, our study can also identify the candidate proteins which play key role in cold acclimation in jojoba seedlings, which should facilitate the understanding of the low temperature stress response in jojoba at the molecular level.

## 2. Results

### 2.1. Physiological Response of Jojoba Seedlings to Cold Stress

**Figure 1:**
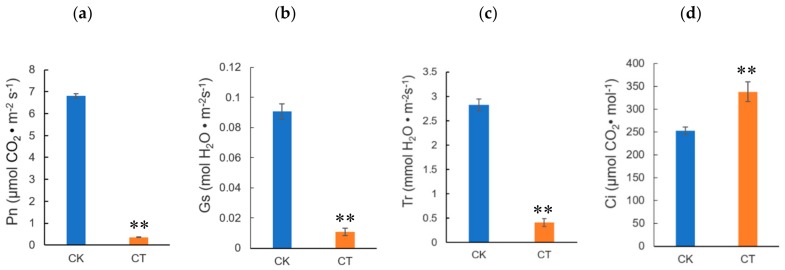
Cold stress-induced changes in photosynthesis related parameters in jojoba leaves from cold stress-treated group (CT) and control group (CK). (**a**) net photosynthesis rate (Pn); (**b**) stomatal conductance (Gs); (**c**) transpiration rate (Tr); (**d**) intercellular carbon dioxide concentration (Ci). Data were represented as means ± SD from five biological replicates (* *p* < 0.05, ** *p* < 0.01).

**Figure 2:**
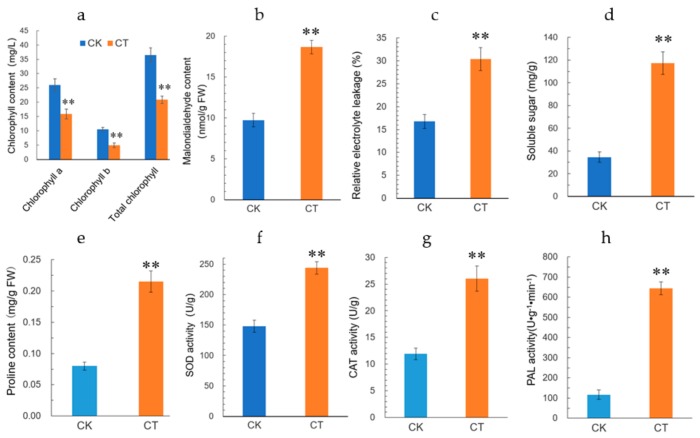
Cold stress-induced alterations of physiological parameters in jojoba leaves from cold stress-treated group (CT) and control group (CK). (**a**) chlorophyll content; (**b**) malondialdehyde (MDA); (**c**) relative electrolyte leakage (REL); (**d**) content of soluble sugars; (**e**) proline content; (**f**) activity of superoxide dismutase (SOD); (**g**) activity of catalase (CAT); (**h**) activity of phenylalanine ammonia-lyase (PAL). Data were represented as means ± SD from five biological replicates (* *p* < 0.05, ** *p* < 0.01).

To investigate the physiological changes in jojoba leaves exposed to cold condition, the jojoba seedlings were treated with non-lethal cold treatment and several physiological and biochemical parameters were measured. Firstly, as expected, the physiological status of the jojoba was affected by cold stress and after cold treatment, the color of jojoba leaves changed from green to gray-green (Figure S1). The retarded growth typically induced by cold stress might be associated to the impaired photosynthesis in jojoba seedlings under cold stress conditions (Figure 1) and change of leaf color may result from the decreased chlorophyll content in jojoba leaves (Figure 2a).

Cold stress is expected to promote the membrane peroxidation, resulting in the elevated level of plasma membrane permeability. Malondialdehyde (MDA) and relative electrolyte leakage (REL) can be used to evaluate the plasma membrane lipid peroxidation and integrity, respectively. In the present study, both MDA content and REL level in jojoba leaves were increased significantly under cold stress (Figure 2b,c), indicating that the treatment regimen we used caused plasma membrane damage in jojoba leaf. Osmotic homeostasis may be disturbed under cold stress and we determined the cold stress induced changes in soluble sugars and proline in jojoba leaves (Figure 2d,e). The levels of soluble sugars and proline were increased significantly under cold stress. We speculated that the accumulation of soluble sugars and proline in jojoba leaves probably help to maintain osmotic balance during adaptation to the cold stress.

Reactive oxygen species (ROS) always accumulated in stressed plants and the activities of antioxidant enzymes will be regulated correspondingly. In cold-stressed jojoba leaves, the activities of superoxide dismutase (SOD) and catalase (CAT) were up-regulated (Figure 2f,g) and these antioxidant enzymes probably contribute to ROS scavenging in cold-stressed jojoba leaves. In addition, the activity of phenylalanine ammonia-lyase (PAL), a key enzyme catalyzes the first metabolic step from primary metabolism to the secondary phenylpropanoid metabolism, was observed to up-regulated in cold-stressed jojoba leaves (Figure 2h).

### 2.2. iTRAQ Analysis and Identification of Differentially Accumulated Proteins

To investigate the proteomic changes associated with cold stress exposure in leaves of jojoba seedlings, iTRAQ analysis was conducted to identify the differentially accumulated proteins (DAPs) between the control and cold-treated plants. High-resolution LC–MS/MS was employed to detect and quantitate proteins in the jojoba leaves. The protein concentration of protein samples was measured by BCA method and the quality of each protein samples were evaluated by (polyacrylamide gel electrophoresis) SDS-PAGE analysis (Figure S2).

After labeling, the combined iTRAQ labeled peptides were fractionated by strong cation exchange (SCX) chromatography (Figure S3). The mass spectrometry proteomic data of the present study have been deposited in the PRIDE PRoteomics IDEntifications (PRIDE) database under the database identifier PXD007063.

**Figure 3:**
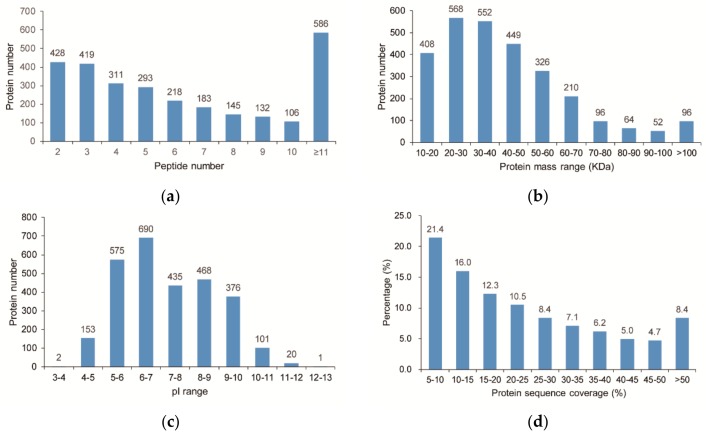
Characteristics of the identified unique proteins in jojoba leaf samples. (**a**) Unique peptide number distribution; (**b**) Protein mass distribution; (**c**) Protein isoelectric point distribution; (**d**) Peptide coverage of the identified proteins.

A total of 23,422 unique peptides (FDR ≤ 0.01) were obtained and 2821 proteins were ultimately identified. The distribution of peptide number is shown in Figure 3a and all of the identified proteins having at least two peptides. The predicted molecular weights and isoelectric points (pIs) of the various identified proteins also showed high degrees of variation (Figure 3b,c), with molecular weights ranging from 10.3 to 254.6 kDa with a median of 37.8 kDa and pIs ranging from 3.95 to 12.06 with a median of 6.98. Moreover, most of the identified proteins have good peptide coverage (Figure 3d).

Of all the detected proteins, 2821 appear in each replicate of all samples and the relative quantifications of these proteins were used for further analyses. Statistical t-test analysis was used to identify the candidate proteins that are involved in the cold stress response in jojoba leaves. Of the 2821 proteins that were quantitated, a total of 109 unique proteins showed differential accumulation pattern (Table S2). Among these DAPs, 31 were up-regulated under cold stress, while the other 78 DAPs were down-regulated.

### 2.3. Functional Annotation and Classification of the Differentially Accumulated Proteins

To understand the biological roles of the DAPs in response to cold stress in jojoba leaves, we annotated the DAPs by the enrichment analysis in the Gene Ontology (GO) function term and the Kyoto Encyclopedia of Genes and Genomes (KEGG) pathway.

**Figure 4:**
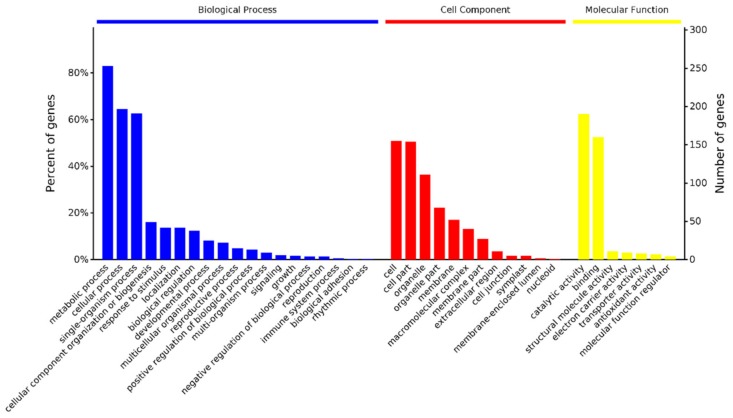
GO functional classification of the identified DAPs.

The amino acid sequences of the 109 DAPs were extracted from customized jojoba protein database based on their ID, then blastp algorithm was performed against the GO and KEGG databases. A total of 1215 GO terms and 38 KEGG terms were identified with a *p* value < 0.05. The top 3 categories of Biological Process terms were metabolic process, cellular process and single-organism process, the top 3 class of cellular component terms were cell, cell part and organelle and the top 3 categories of molecular function terms were catalytic activity, binding and structural molecule activity (Figure 4). The major KEGG pathways included biosynthesis of secondary metabolites (9 DAPs), Carbon fixation in photosynthetic organisms (6 DAPs), phagosome (7 DAPs), Ribosome (5 DAPs), oxidative phosphorylation (4 DAPs), protein processing in endoplasmic reticulum (3 DAPs) and phenylpropanoid biosynthesis (3 DAPs).

GO enrichment analyses of the DAPs using agriGO [16] revealed 57 enriched GO terms. These enriched GO terms were associated with various biological processes, including response to abiotic stimulus, phenylpropanoid biosynthetic process and carbohydrate metabolic process. The overrepresented GO cellular component terms included cytoplasm, chloroplast and ribosome. The top 3 enriched GO molecular function terms were catalytic activity, lyase activity and structural molecule activity.

**Figure 5:**
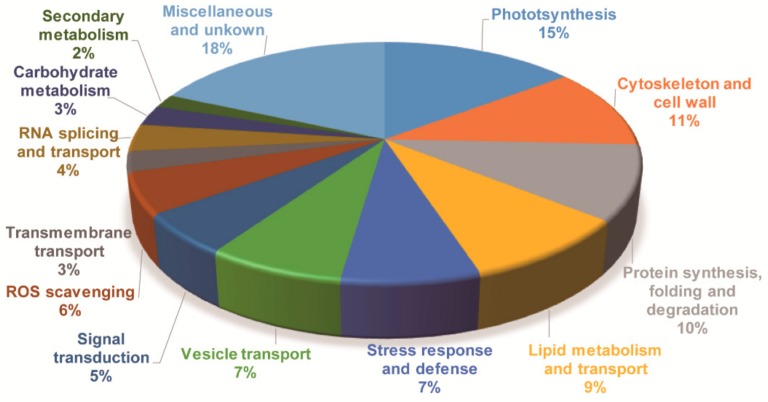
Functional categorization of the differentially accumulated proteins in jojoba leaves under cold stress.

Although GO and KEGG analysis can provide similar and overlapping results, integrating these results will help to reveal more accurately the biological processes represented by the DAPs and their biological significance. All DAPs were also annotated by aligning to Arabidopsis protein database (TAIR10) and Swiss-Prot database. Based on the annotation results, together with results of the GO and KEGG analyses, the DAPs were classified into 14 categories according to their putative biological functions, i.e., photosynthesis, cytoskeleton and cell wall, protein synthesis, folding and degradation, lipid metabolism and transport, stress response and defense, signal transduction, RNA splicing and transport, vesicle transport, carbohydrate metabolism, transmembrane transport, ROS scavenging, secondary metabolism and miscellaneous and unknown proteins (Figure 5 and Table S2). Their possible functions in cold stress signaling and response will be discussed later.

### 2.4. Gene Expression Analysis of the Cold Stress Responsive Proteins

**Figure 6:**
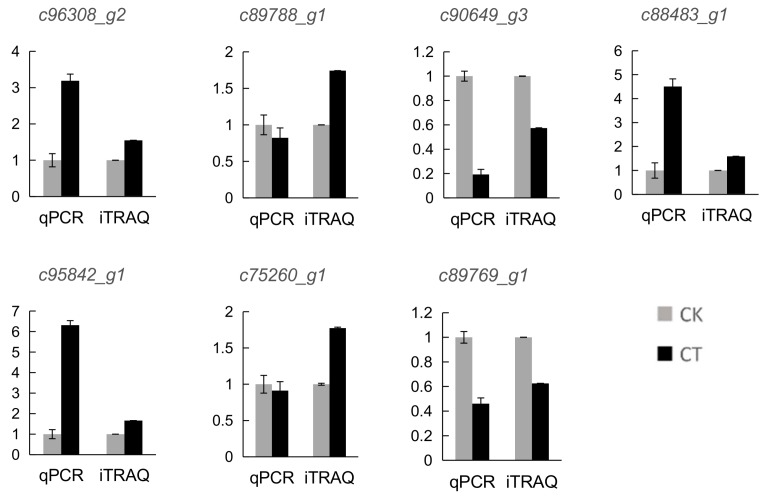
Gene expression analysis of the DAPs and comparison with the change pattern at the protein level revealed by iTRAQ analysis. Values were represented as means ± SD from three biological replicates. CK, control group; CT, cold-treated group.

To validate the results of the quantitative proteomic analysis, 7 DAP coding genes were randomly selected for quantitative real time PCR (qRT-PCR) analysis (Figure 6). The expression levels of most of these genes exhibited the same trend with the protein abundance of the corresponding DAPs. However, the expression level of two genes (c89788_g1 and c75260_g1) showed the opposite change pattern with the abundance of their corresponding proteins. The discrepancy between the transcription level of the DAPs and the abundance of the corresponding proteins have been reported in previous studies [13] and this difference probably resulted from posttranslational modifications of proteins under cold stress, such as protein phosphorylation.

### 2.5. Molecular Network Involved in Cold Stress Response in Jojoba Leaves

**Figure 7:**
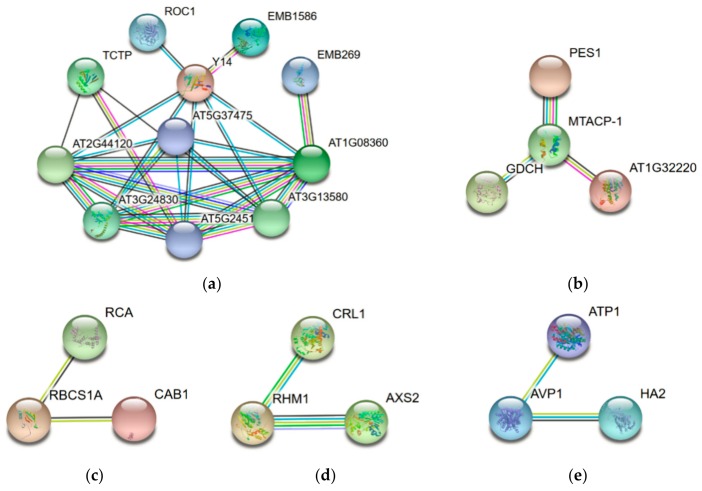
The protein-protein interaction (PPI) network of the differentially accumulated proteins (DAPs) in jojoba leaves under cold stress. Edge color represents protein-protein associations among different proteins: blue for known interactions from curated database, black for predicted interactions base on co-expression, pink for experimentally determined known interactions, green for predicted interactions base on gene neighborhood, red for predicted interactions base on gene fusions, dark blue for predicted interactions base on gene co-occurrence, yellow-green for predicted interactions base on text mining and light blue for predicted interactions base on protein homology. The largest network (**a**) was mainly associated with proteins synthesis and folding, the second largest network (**b**) was related to the mitochondrial respiratory chain. The other subnetworks were associated with photosynthesis (**c**), cell wall (**d**) and transmembrane transport (**e**).

To reveal the interaction networks associated with the cold stress response in jojoba leaves, the protein-protein interaction (PPI) networks were constructed using the STRING protein-protein interaction database (Figure 7). Due to the lack of protein interaction data of jojoba and its closely related species, we used the homologous proteins in *Arabidopsis thaliana* to construct the protein interaction networks. For ease of understanding, the names of the DAPs were represented by the names or the locus numbers of the homologous proteins in Arabidopsis (http://www.arabidopsis.org) in the PPI map.

The largest network (Figure 7a) consists of 11 DAPs associated with proteins synthesis and folding, suggesting the cold stress significantly affected the protein synthesis in jojoba. The second largest network (Figure 7b) consisted of 4 proteins and most of them were related to the mitochondrial respiratory chain. The other subnetworks were associated with photosynthesis (Figure 7c), cell wall (Figure 7d) and transmembrane transport (Figure 7e). In sum, most of the cold stress-regulated biological processes identified via functional annotation and classification analyses were also highlighted in the PPI map.

## 3. Discussion

Our previous EST analysis had identified several candidate genes which may be involved in the water-deficient stress response in jojoba plants [17]. In the present proteomic analysis, a large number of cold stress-responsive proteins were identified in jojoba leaves. As expected, some well-known stress-inducible proteins were found, such as copper/zinc superoxide dismutase 1 (CSD1) and cold-regulated 47 (COR47). Some cold-responsive proteins reported previously in other plant species were also presented in the list of the DAPs, including ferredoxin 3 (FD3) and PHE ammonia lyase 1 (PAL1) [18]. These results support the reliability and robustness of the iTRAQ technology in investigating the plant response to environmental stress. Our data showed that several proteins were up-regulated significantly under cold stress and some of them might play important roles in the response to cold stress in jojoba. The possible biological functions of these DAPs in cold stress adaptation are further discussed below.

### 3.1. Proteins Involved in Stress Signal Transduction

In plant cells, perception of extracellular stimuli was mediated by the plasma membrane receptors and transduced by signaling pathway. In the present study, several components in Ca^2+^ and abscisic acid (ABA) signaling were regulated under cold stress, highlighting their pivotal roles in the jojoba’s response to cold stress.

Ca^2+^ plays an essential role in plant cells in response to environmental stimuli as a second messenger and Ca^2+^ concentration has been found to increase in response to cold stress [19]. In our study, two calcium-binding proteins, i.e., calcium-binding EF-hand family protein and annexin 4, were found to be down-regulated under cold stress. Although calcium ion has been demonstrated to play an important role in the low temperature perception and signaling, in the present study, we can still find some components of calcium signaling pathway changes in abundance after 7 days of cold stress, indicating that the calcium ion signaling pathway may also be involved in low temperature adaptation in jojoba.

ABA signaling plays important role in stress response in plant and as expected, several DAPs involved ABA signaling were identified, including Serine/threonine-protein kinase GRIK2 (GRIK2), CPCK2 (chloroplast localized subunit of casein kinase 4) and annexin 4. SNF1-RELATED PROTEIN KINASE 1.1 (SnRK1.1) is a key component in abscisic acid-activated signaling pathway and Arabidopsis GRIK1 specifically activates SnRK1.1 by phosphorylation of its activation-loop [20]. Annexin 4 has been shown to play a vital role in abscisic acid signal transduction in Arabidopsis in a Ca^2+^-dependent manner [21].

In addition, several DAPs involved synthesis and signaling of other phytohormones were also identified. Of them, ethylene-forming enzyme (EFE) is an enzyme involved in the ethylene biosynthesis and EXORDIUM like 5 (EXL5) is involved in brassinosteroid-dependent regulation of growth and development [22]. These data indicated that multiple phytohormone signaling pathways were adjusted in jojoba to adapt to the cold condition.

### 3.2. Proteins Involved in Photosynthesis and Carbohydrate Metabolism

Photosynthesis is greatly inhibited by low temperature in various plant species, especially for cold sensitive tropical crops such as jojoba. We observed that the physiological status of jojoba seedlings was affected by the cold stress treatment. The effect might relate to the decrease of the net photosynthesis as revealed by photosynthetic performance measurement (Figure 1).

Sixteen DAPs involved in photosynthesis were regulated by cold stress. In line with the impaired photosynthesis, most of the photosynthesis related DAPs were down-regulated in abundance, including three ribulose bisphosphate carboxylase small chain proteins and a Ribulose bisphosphate carboxylase/oxygenase activase. The only three up-regulated DAPs were FD3, NADP-malic enzyme 4 (NADP-ME4) and glyceraldehyde-3-phosphate dehydrogenase of plastid 1 (GAPCP-1). Ferredoxins are small, soluble iron-sulfur proteins that deliver electrons in many metabolic reactions and the chloroplast localized ferredoxins mainly function as electron transfer proteins to transfer reducing equivalents from photosystem I (PSI) to NADPH during linear electron flow (LEF). Ferredoxins were down or up-regulated under abiotic stress in several plant species such as rice and maize [18] and expression of a sweet pepper ferredoxin enhanced the tolerance to heat stress in *Arabidopsis thaliana* [23]. In the present study, the up-regulation of FD3 may help to prevent photo-oxidative damage under cold stress through cyclic electron flow (CEF) in jojoba leaves.

We observed significant increase in soluble sugars and proline level in jojoba leaves under cold stress. These molecules not only function as osmoprotectants but also protect the membrane via the interaction with the lipid biolayer and high levels of sugars inhibit photosynthesis in plant under cold stress [24]. Considered that the photosynthesis is greatly inhibited by cold stress, the accumulation of soluble sugars probably resulted from enhanced starch degradation, which was consistent with observations in tea plant [25]. As expected, many enzymes affecting sugar content were regulated under cold stress and these enzymes included a trehalose-6-phosphatase synthase s7, which is involved in trehalose biosynthesis [26] and two enzymes involved in starch synthesis, glucose-1-phosphate adenylyltransferase family protein (AGPase large subunit 3, APL3) and granule bound starch synthase 1) (GBSS1) (Table S2). ADP-Glucose pyrophosphorylase (AGP) catalyzes the rate limiting step in starch biosynthesis and AGPase large subunit 1 (APL1) and AGPase small subunit (APS1) are abundant in photosynthetic tissues and play the dominant role in leaves. APL3 is a large subunit isoform of AGP and present mainly in root [27]. The up-regulation of APL3 in cold-stressed jojoba leaves suggested that APL3 might also play a role in starch synthesis in leaves when the plants were exposed to cold stress. GBSS1 is the only starch synthase isoform required for amylose synthesis in chloroplast [28] and the up-regulation of GBSS1 in cold-stressed jojoba indicates that the proportion of amylose in starch may increase in cold-stressed jojoba leaves. In brief, our data showed that reorganization of starch metabolism was an essential process for jojoba to survive under low temperature conditions.

### 3.3. Proteins Involved in ROS Scavenging

Dysfunction of the photosynthetic apparatus under cold conditions exposes the plant to photoinhibition and can lead to elevated levels of ROS. In the present study, 6 DAPs involved in ROS scavenging were identified. Glutathione (GSH) plays key role in cell redox homeostasis. Three of these DAPs were involved in regulating GSH concentration, including GLYOXALASEI 6 (GLYI6) and two glutathione *S*-transferase family proteins. These results indicated that GSH metabolism was adjusted in jojoba leaf cells upon cold stress. Among these DAPs, glyoxalase Is (GLYIs) are one of the two groups of enzymes forming glyoxalase pathway and glyoxalase pathway has been shown to play an important role in stress tolerance. GLYI uses GSH as a cofactor for the detoxification of methylglyoxal (MG), high level of which is toxic to cells [29].

CSD1 were found to be up-regulated significantly in jojoba leaves under cold stress. In line with the change in protein abundance, the superoxide dismutase activity was elevated in cold stress jojoba leaves (Figure 2f). CSD1 encodes a cytosolic copper/zinc superoxide dismutase and its expression is negatively regulated by miR398. SODs catalyze the dismutation of superoxide into oxygen and hydrogen peroxide, constitute the first line of defense against ROS in cell. CSD1 has been observed to up-regulated under stressful conditions in many plant species [18,30] and transgenic plants that express CSD1 have shown enhanced tolerance to multiple stresses [30]. The up-regulation of CSD1 might contribute to cold acclimation in jojoba by repressing the elevation of ROS level.

### 3.4. Proteins Involved in Stress Response and Defense

Eight proteins involved in stress response and defense were identified as DAPs in the present study. Several proteins of these category, i.e., COR47, cystatin B, ARABIDOPSIS THALIANA KUNITZ TRYPSIN INHIBITOR 5 (ATKTI5), MLP-like protein 34 (MLP34), were frequently identified as differentially accumulated proteins under stressful conditions in previous proteomic studies in other plant species [18].

It is noteworthy that almost all DAPs involved in stress response and defense were down-regulated under cold stress in jojoba leaves and the only cold-induced DAPs in this category is COR47. Dehydrin (DHN) is a large family of proteins present in plants and DHNs are produced in response to environmental stresses. COR47 is one of DHNs that accumulate during the abiotic stress such as drought, salinity, freezing, or by treatment with ABA. COR47 is one the principal DHNs that accumulate under low temperature stress in *A. thaliana* and overexpression of COR47 improved cold stress tolerance of *A. thaliana* seedling [31]. COR47 is regarded as the marker genes of CBF/DREB pathway during cold acclimation, thus the up-regulation of COR47 under cold exposure probably play important role in cold adaptation of jojoba seedlings.

### 3.5. Proteins Involved in Cell Wall Modification and Osmotic Homeostasis

Cold stress was previously reported to affect the cell walls in pea seedlings [32] and cell wall modification is essential for plant acclimation to environmental stresses [33]. As expected, several enzymes involved in cell wall modification were differentially accumulated in cold-stressed jojoba leaves. Among these DAPs, pectin methylesterase CGR2 (CGR2) functions in the modification of cell walls via methylesterification of cell wall pectin [34]. Lignin is an important component of cell walls and several genes involved in lignin biosynthesis, i.e., PAL1, CCR (cinnamoyl CoA: NADP oxidoreductase)-LIKE 1 and a class III peroxidases, peroxidase 52, were identified as DAPs, indicating lignin synthesis was regulated under cold stress. Among these DAPs, PAL1 was up-regulated significantly and the PAL enzyme activity measurement validated the up-regulation of PAL at the protein level. PAL1 catalyzes the first step in the phenylpropanoid pathway [35] and another important enzyme in the phenylpropanoid pathway, flavonol synthase 1 (FLS1) was also up-regulated in cold-stressed jojoba leaves. In addition to act as the precursors for lignin synthesis, derivatives of phenylpropanoid pathway have various biological functions in plants, for example, some flavonoids function as protectants against oxidative stress induced by abiotic stress or pathogen attack [36].

Cold stress can induce inhibition of water uptake and indirectly resulted in osmotic stress in cells [37], thus, maintenance of the cell’s osmotic potential under cold conditions is one of the major challenges for plant growth and development. In the present study, the increased soluble sugars in cold-stressed jojoba leaves can help the cell to lower water potential in cytoplasmic matric (Figure 2d). At the same time, several aquaporins and ion transporters were found to be differentially accumulated under cold stress and these DAPs included plasma membrane intrinsic protein 3 (PIP3), inorganic H pyrophosphatase family protein AVP1 (AVP1) and PLASMA MEMBRANE PROTON ATPASE 2 (PMA2). Of these DAPs, PMA2 probably contributes to the H^+^-electrochemical potential difference across cytoplasma membrane that drives the active transport of nutrients by H^+^-symport and PMA2 is also shown to be involved in cell expansion, by acidifying the apoplasm and thus, activating proteins involved in loosening the cell wall like expansins [38]. In the present study, the decreased PMA2 may be associated to the cell wall rigidification induced by cold stress [39]. AVP1 plays an important role in trans-tonoplast membrane proton gradient that is used to energize secondary transporters like vacuolar Na^+^/H^+^ antiporters and ectopic expression of an Arabidopsis AVP1 improves drought- and salt tolerance in cotton [40]. The up-regulation of AVP1 in cold-stressed jojoba leaves might contribute to the osmotic homeostasis of the cells.

Under the low temperature environment, the cell volume might become smaller. To cope with such stress, the cytoskeleton, cell wall and plasma membrane would change correspondingly. We found five cytoskeleton-related proteins differentially accumulated under cold stress. These DAPs included tubulin alpha-4 chain (TUA4), tubulin beta 8 (TUB8) and actin 1 (ACT1). The cytoskeleton-related DAPs probably participate in modulating the cytoskeleton organization in cold-stressed jojoba leaf cells.

### 3.6. Proteins Involved in Protein Synthesis, Folding and Degradation

Cold stress seriously affected the protein synthesis and folding and previous proteomic studies have identified many stress-responsive proteins associated with protein synthesis, folding and degradation in plants [18]. In jojoba, 11 DAPs involved in protein metabolism were identified, including 6 involved in translation, 3 in protein folding and 2 in protein modification. Extensive protein interactions were predicted among the DAPs in this category (Figure 7a).

Although most of the DAPs involved in protein metabolism were down-regulated, some of them were induced under cold stress. For example, 60S ribosomal protein L7-3 and L10a-1 and an eukaryotic translation initiation factor 3 (eIF-3) subunit were down-regulated, while 60S ribosomal protein L7-4 and L13a-2 were increased in abundance under cold stress. These data indicated that the translation apparatus was adjusted to adapt to cold environment.

Besides 60S ribosomal protein L7-4 and L13a-2, two cold-induced DAPs, i.e., signal peptidase complex catalytic subunit SEC11C (SEC11C) and staurosporin and temperature sensitive 3-like a (STT3A), were identified in jojoba leaves under cold stress. Signal peptidase is a group of enzymes help to remove signal peptides from nascent proteins Since no stress responsive signal peptidase have been reported by now, the biological significance of the up-regulation of SEC11C observed in the present study is still to be investigated.

Environmental stresses often lead to the accumulation of misfolded proteins in the endoplasmic reticulum (ER) lumen. Besides molecular chaperone and peptide disulfide isomerase, which help the refolding of the misfolded proteins, *N*-glycosylation in ER has been shown to regulate protein quality control. Protein *N*-glycosylation in ER is catalyzed by a multi-subunit enzyme, the oligosaccharyltransferase (OST) complex. One of the cold-induce DAPs in the present study, STT3A, is one of the catalytic subunits of OST. Previous studies showed that STT3a is required for recovery from the unfolded protein response and for cell during salt/osmotic stress recovery [41]. Our results indicated that cold stress induced STT3a possibly contribute to the cold acclimation of jojoba seedling by participated in protein folding and strengthening protein quality control in ER.

### 3.7. Proteins Involved in Lipid Metabolism and Transport

Lipids are involved in a wide variety of physiological processes in plant cells. For example, lipids form the cytoplasma membrane and membrane of organelles, protect tissues by forming leaf cuticle and wax; lipids participate in the photosynthetic capture of light in chloroplast and function as signaling molecules in stress signal transduction [42]. In the present study, 10 DAPs involved in fatty acid metabolism and transport were identified, indicating a significant change were taken place in lipid metabolism in cold-stressed jojoba leaves and such a phenomenon was reported in Arabidopsis and *Eutrema salsugineum* [42]. There of these DAPs, i.e., glycosylphosphatidylinositol-anchored lipid protein transfer 6 (LTPG6), fatty acid export 1 (FAX1) and ROSY1 are involved in lipid and sterol transfer. Among them, ROSY1 has been shown to be involved in the regulation of gravitropic response and basipetal auxin transport in roots [43] and the down-regulation of ROSY1 in jojoba leaves suggested its possible role in cold stress response. The remaining 8 DAPs in this category were mainly associated with lipid synthesis and catabolism. Among them, 3-DEOXY-D-ARABINO-HEPTULOSONATE-7-PHOSPHATE 2 (DAHP2) catalyzes the first step of the shikimate pathway, a key pathway for the synthesis of aromatic primary and secondary metabolites [44]. The up-regulation of DAHP2 in cold-stressed jojoba leaves indicated that more carbon flux was used to synthesize lipid and secondary metabolites. Mitochondrial acyl carrier protein 1 (MTACP-1), a member of the mitochondrial acyl carrier protein (ACP) family, is involved in fatty acid and lipoic acid synthesis in mitochondria [45]. Cytochrome B5 isoform B and E are membrane bound hemoproteins and are involved in oxidation-reduction process by functioning as electron carrier for some membrane bound oxygenases like fatty acid desaturases [46].

When plants were exposed to environmental stresses, the thylakoid membranes in chloroplasts are disintegrated and galactolipid are broken down, leading to the accumulation of phytol and free fatty acids, which are toxic to cells. Theses phytol and fatty acids can be converted into fatty acid phytyl esters and triacylglycerol by phytyl ester synthase (PES) [47]. PES1 was up-regulated in jojoba seedling under cold treatment, which would help to maintain the integrity of the photosynthetic membrane during cold stress. Thus, the up-regulation of PES1 probably play a vital role in cold adaptation to cold conditions for jojoba.

## 4. Materials and Methods

### 4.1. Plant Growth and Cold Stress Treatment

Jojoba seedlings were grown in commercial pots at 25/20 °C (*d*/*n*) under a photosynthetic photon flux density of 150 μmol m^−2^ s^−1^ with long-day conditions (16/8 h light/dark cycle) for 1 month. Thirty female Jojoba seedlings were divided into two groups randomly and all the seedling have similar height and 7–8 pairs of true leaves. The first group was still grown in tissue culture room for additional 7 days and served as the control group. The second group were transferred to the cold-stress conditions (150 μmol m^−2^ s^−1^ light intensity, 20/15 °C for the first two days and 15/10 °C *d*/*n* for the remaining five days). A growth chamber Pervical LT-36VL (Percival Scientific, Inc., Perry, IA, USA) was used for plant growth and cold treatment. After 7 days of treatment, the fifth and sixth pairs of leaves in the both groups were collected. Part of these leaves were used for biochemical and physiological parameter measurement and the remaining leaves were frozen immediately in liquid nitrogen and then stored at −70 °C for further RNA and protein extraction. Each sample were pooled from three individual plants and each group had at least three biological replicates.

### 4.2. Physiological and Biochemical Parameter Measurements

Measurements of photosynthesis, stomatal conductance, intercellular carbon dioxide concentration and transpiration rate were conducted using a portable gas analysis system, LI-COR 6400 (LICOR Inc., Lincoln, NE, USA). The chlorophyll contents were determined using a UV-vis spectrophotometer according to a previously described method [48]. MDA content and REL were measured using the previous described methods [49]. The content of soluble sugars was measured using the anthrone method [50]. Proline concentrations were measured in a UV-vis spectrophotometer by using the ninhydrin reaction method [51]. For enzyme activity measurement, five hundred milligram fresh leaves were homogenized with 5 mM phosphate buffer (pH 7.0) containing 1 mM EDTA and 2% PVPP at 4 °C. After centrifugation (12,000 *g*) for 15 min at 4 °C, the supernatant was collected and used for enzymes measurement. The activities of CAT and SOD were conducted according to the protocol provided by the manufacturer (Nanjing Jiancheng Institute of Biotechnology, Nanjing, China). PAL activity was assayed using the method of McCallum and Walker [52]. Data are presented as means ± standard deviation (SD) from five independent biological replicates.

### 4.3. Protein Extraction

Leaf samples from cold stress-treated seedlings (CT) and unstressed seedlings (CK) were ground into fine powder with liquid nitrogen. The power was suspended in an acetone solution containing 10% trichloroacetic acid and 65 mM Dithiothreitol (DTT). After thoroughly mixing by vortexing, proteins were precipitated at −20 °C for 1 h. Proteins were then harvested by centrifuging at 10,000 rpm (Eppendorf 5430R; Eppendorf Ltd., Hamburg, Germany) at 4 °C for 50 min. The resulting supernatant was removed and the protein pellet was washed three times with cold acetone and then dried by lyophilization. The pellet was then suspended in STD buffer (4% SDS, 1 mM DTT, 150 mM Tris-HCl, pH 8.0) and mixed thoroughly. After 5 min incubation in boiling water, the suspensions were dispersed by ultrasonication (80 w: 10 times for 10 s each, with 15 s intervals). After incubated in a boiling water bath for 5 min, the final protein solutions were collected by centrifugation. The protein concentration was measured using the bicinchoninic acid protein assay kit (Beyotime, Shanghai, China). In addition, the quality of the protein samples was further inspected by SDS-PAGE electrophoresis.

### 4.4. Protein Digestion and ITRAQ Labeling

Protein digestion was conducted using the FASP method [53]. The peptide content was determined by spectra density using UV absorption at 280 nm. The peptide mixture was labeled with the 8-plex iTRAQ reagent according to the manufacturer’s recommendation (AB SCIEX) (113 to 115 tags for the three biological replicates of control group, 116 to 118 tags for the three biological replicates of cold stress-treated group) and vacuum dried.

### 4.5. Strong Cation Exchange (SCX) Chromatography and LC–MS/MS

All the labeled peptide samples were mixed together and then fractionated by strong cation exchange (SCX) chromatography using an AKTA Purifier system (GE Healthcare, Waukesha, WI, USA). The collected fractions (33 fractions) were finally combined into 15 pools and desalted on C18 Cartridges (66872-U, Sigma, St Louis, MO, USA).

Peptides separation was conducted with an automated Easy-nLC1000 system coupled to a Q-Exactive mass spectrometer (Thermo Finnigan, San Jose, CA, USA). The peptides were loaded onto a Thermos scientific EASY column (2 cm × 100 μm, 5 μm-C18) equilibrated with 95% Buffer A (Buffer A, 0.1% formic acid) and then the peptides were loaded and separated on a C18 column (75 mm × 250 mm, 3μm-C18) at a flow rate of 250 nL/min. The peptides were separated with an elution buffer B (0.1% formic acid in 84% acetonitrile) gradient as follows: 0–50% for 100 min, 50–100% for 8 min and 100% for 12 min. The Q-Exactive (Thermo Finnigan, San Jose, CA, USA) mass spectrometer was used to collect data in the positive ion mode according to the method described previously [13].

### 4.6. Protein Identification and Quantification

Raw MS/MS data were interpreted with Proteome Discoverer (version 1.4, Thermo Fisher Scientific, Waltham, MA, USA) using the search engine Mascot (Version 2.2, Matrix Science, London, UK) against the customized protein database of jojoba (47,062 sequences, translated from transcriptome sequencing of jojoba leaves and young fruits) and the decoy database. The Mascot parameters were set as follows: enzyme, trypsin; mass values, monoisotopic; peptide mass tolerance, ±20 ppm; MS/MS tolerance, 0.1 Da; max missed cleavages, 2; fixed modifications, Carbamidomethyl (C), iTRAQ8plex (N-term), iTRAQ8plex (K); variable modifications, Oxidation (M). The reverse of the target database was used as the decoy database. False discovery rate (FDR) of both proteins and peptides identification was not more than 1%. Protein identification was supported by a minimum of two unique peptide identification and an amino acid coverage ≥ 5%.

The intensity of the reporter ions from analyzed fragmentation spectrums was used for peptide quantification and the relative quantity was calculated using the Proteome Discoverer 1.4 software according to the user’s guide. Each confident protein quantification required at least two unique peptide and the quantification values were rejected if not all quantification channels are present. The relative quantification of proteins in each group was based on the strength of the reporter ion. The average value of channels of control group was used as internal reference. Ratio was used to assess the fold changes in the abundance of the proteins identified in cold-treated group versus control group. Student’s *t*-test was used to identify significant (*p*-value < 0.05) differences in means between cold-treated and control plants. To be identified as differentially accumulated protein (DAP), a protein must pass *t*-test with *p*-value < 0.05 and with a change ratio > 1.5 or < 0.667.

### 4.7. Function Annotation, Classification of the DAPs

Proteins were functionally annotated by using Blast2GO. GO enrichment analysis was conducted using the singular enrichment analysis (SEA) under agriGO [16] and the Arabidopsis thaliana (TAIR10) were used as backgrounds in combination with Fisher’s test and the Yekutieli multiple-test with a threshold of FDR = 0.05. Subcellular localization information for proteins was collected from UniProt database and TAIR (www.arabidopsis.org). The biological functions of the DAPs were classified based on their gene ontology annotations and their annotation in KEGG (Kyoto Encyclopedia of Genes and Genomes) database (https://www.kegg.jp/). Protein-protein interaction networks were analyzed using the program STRING (http://string-db.org/), a database of known and predicted protein-protein interactions. The confidence score was set at the high level (≥0.900).

### 4.8. Quantitative Real-Time PCR Analysis

Total RNA was extracted from jojoba leaves according to a previously described method [17]. qRT-PCR were performed according to previous descriptions [54]. The gene expressions of selected protein-coding genes were normalized against an internal reference gene, 18S rRNA (NCBI accession number: AF094562). The relative gene expression was determined using the 2^−ΔΔ*C*t^ method [55]. Data are presented as means ± standard deviation (SD) from three independent biological replicates. A *p*-value < 0.05 was determined to be statistically significant. All primers used in the present study are listed in Table S1.

## 5. Conclusions

A combined physiological and quantitative proteomic analysis was performed to investigate the response to cold stress in jojoba, a semi-arid oil producing crop. Our results indicated that cold stress promote the membrane peroxidation and impair the membrane integrity and jojoba leaves respond to cold stress by reducing chlorophyll content and inhibiting photosynthesis, enhancing the ROS scavenging activities via up-regulating the abundance of antioxidant enzymes, adjusting the protein turnover and regulating the abundance of various defense and stress-response related proteins. Ca^2+^ and ABA signaling participated in the stress signaling transduction in the jojoba’s response to the cold stress. Several cold stress induced proteins, including CSD1, COR47, STT3a and PES1 probably play essential roles in jojoba adaptation to the cold stress.

In future studies, we will collect more jojoba low temperature resistant and sensitive varieties, analyze the expression of the low temperature-responsive proteins identified in the present study in these varieties and further identify the protein markers closely associated with the low temperature tolerance in jojoba. We will conduct functional analysis of the low-temperature inducible proteins identified in the present study by expressing these proteins in *Escherichia coli*, yeast and/or *Arabidopsis thaliana* to evaluate their effect on low temperature tolerance of cells.

## Supplementary Materials

The following are available online at http://www.mdpi.com/1422-0067/20/2/243/s1.
