# Supplementary material for: Quantitative Proteomic Analysis of the Response to Cold Stress in Jojoba, a Tropical Woody Crop
Source: Int J Mol Sci. 2019 Jan 9;20(2):243. doi: 10.3390/ijms20020243 (PMC6359463; doi:10.3390/ijms20020243)
Supplement: Supplementary file 1 [file ijms-20-00243-s001.zip › supplementary material_0101.docx]

| a | b |
| --- | --- |
| 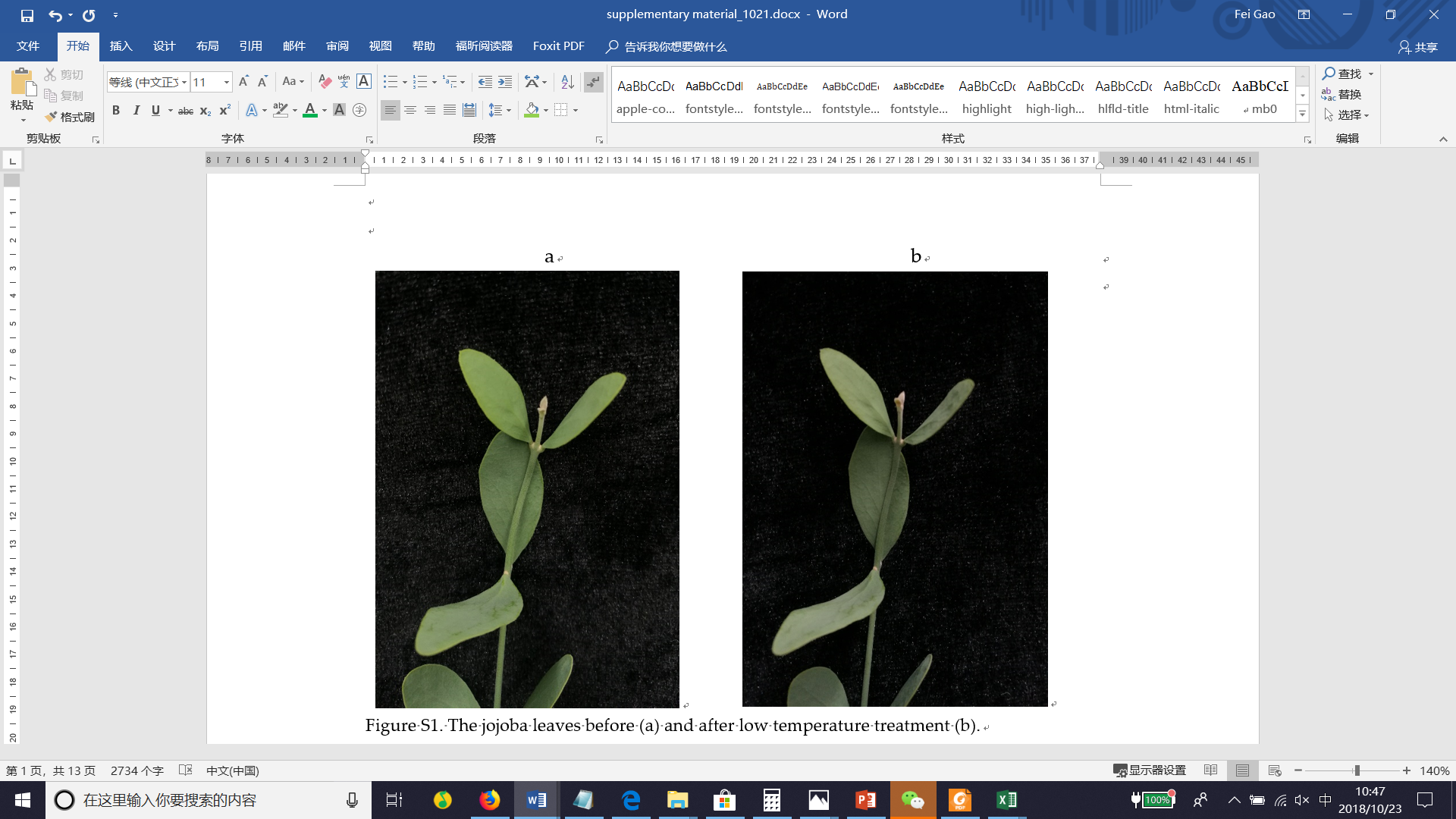 | 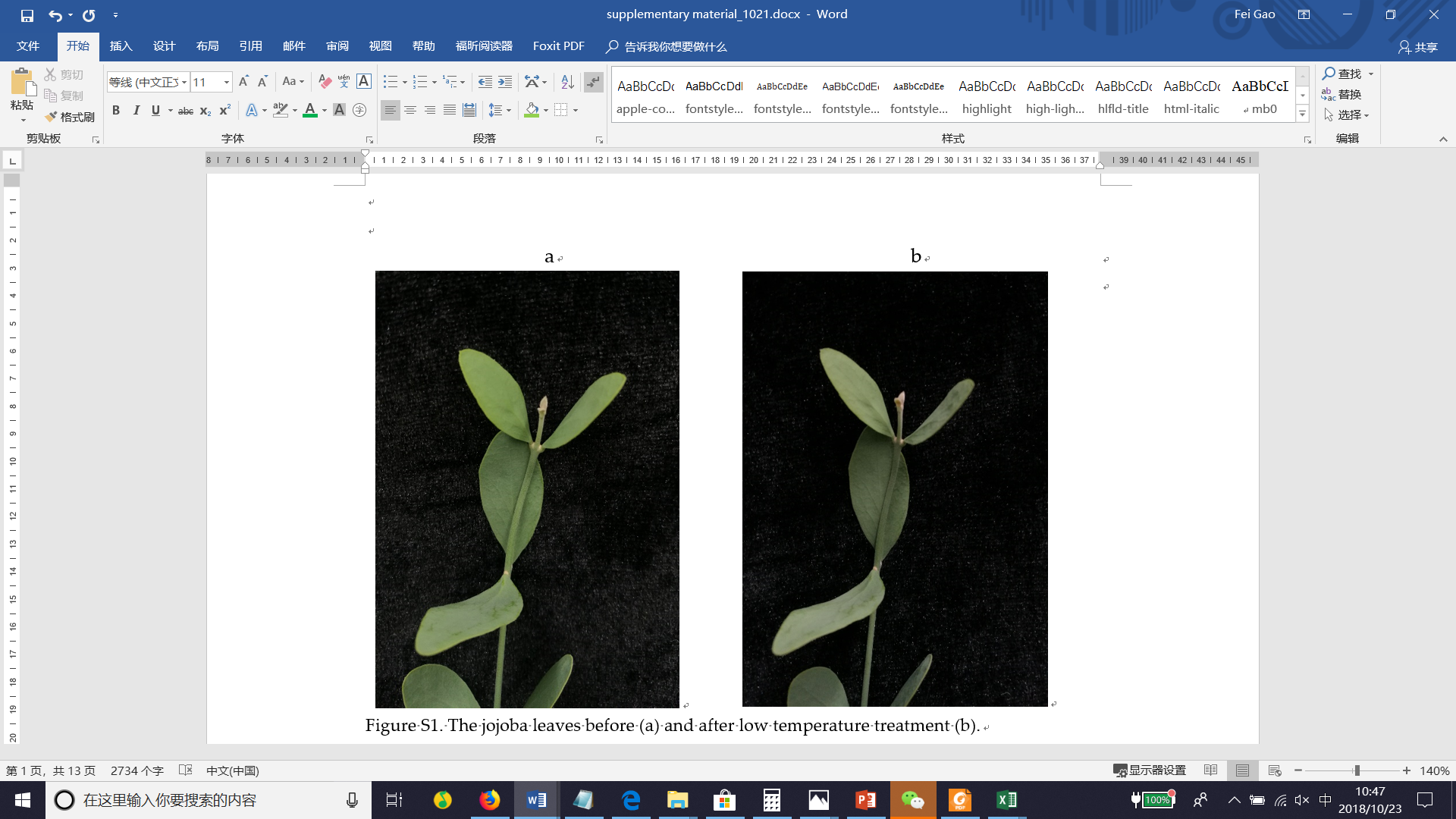 |

Figure [S1](http://journal.frontiersin.org/article/10.3389/fpls.2016.00136/full#SM1). The jojoba leaves before (a) and after cold stress treatment (b).

Marker CK1 CK2 CK3 CK4 CT1 CT2 CT3 CT4


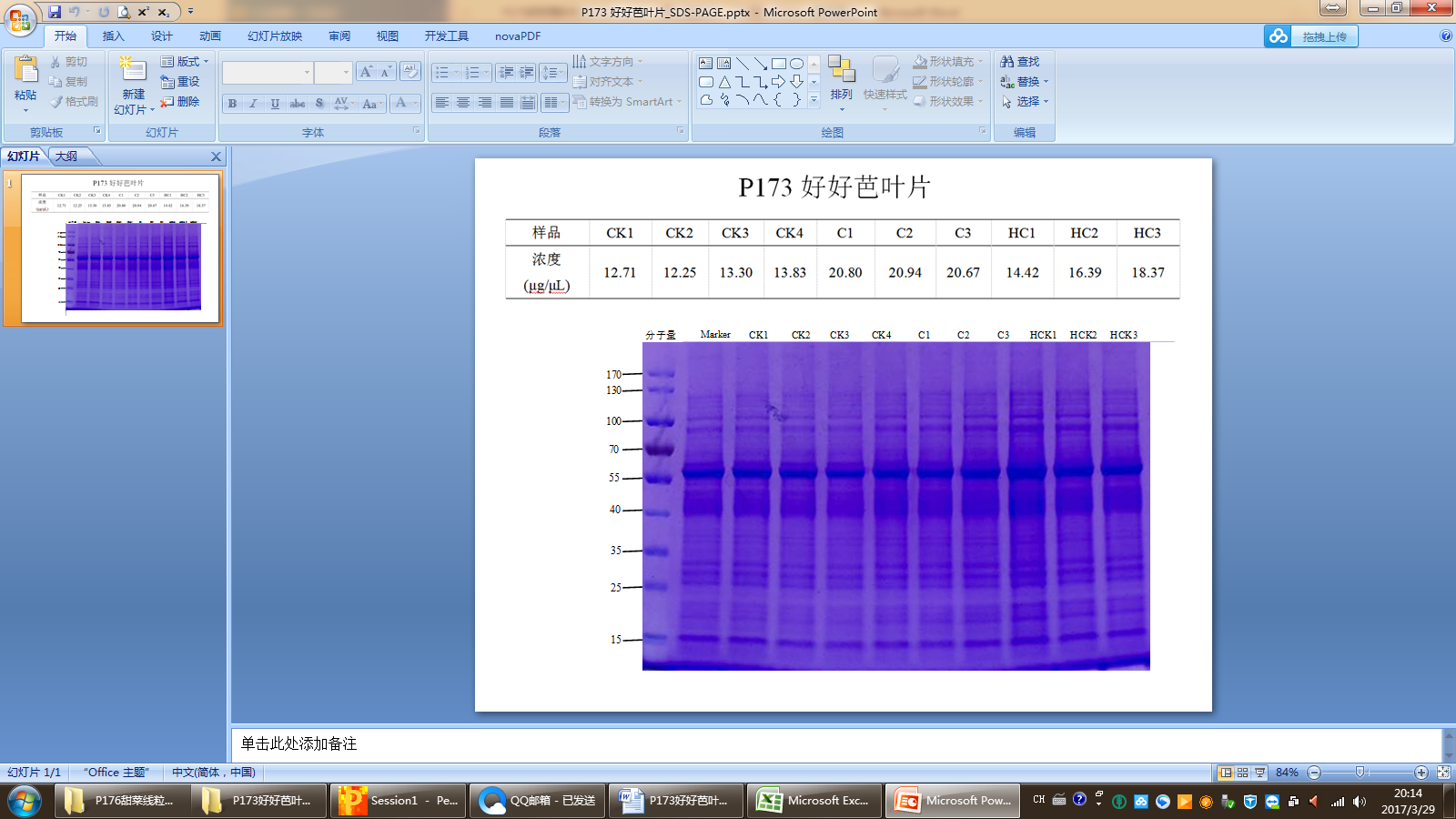


Figure S[2](http://journal.frontiersin.org/article/10.3389/fpls.2016.00136/full#SM1). SDS-PAGE analysis of the leaf protein samples (CK1-3 and CT1-3 were used for iTRAQ analysis). Protein samples (20μg) were separated on 12.5% SDS-PAGE electrophoresis.


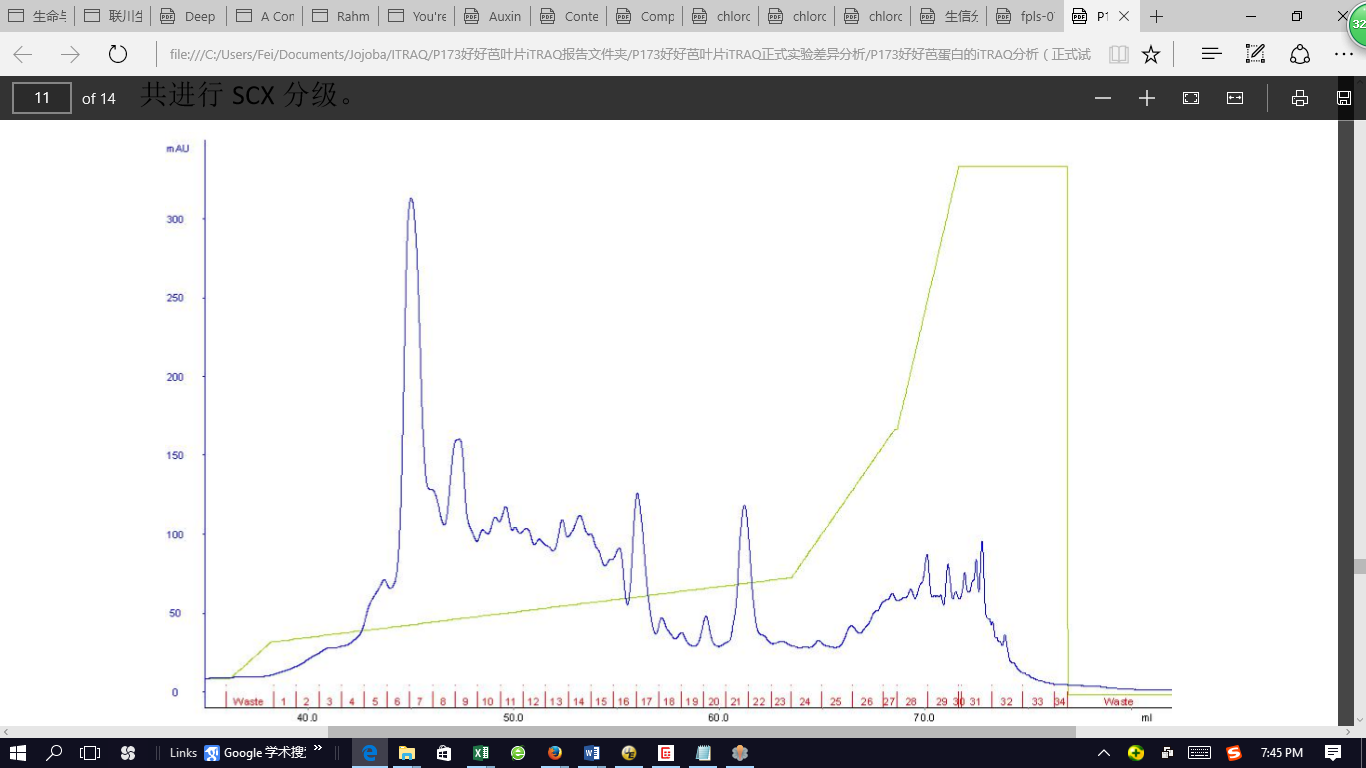


Figure S3 The iTRAQ labeled peptides were fractionated by strong cation exchange (SCX) chromatography

Table S1 Primers used for qRT-PCR analysis

| Gene ID | Forward primer (5′-3′) | Reverse primer (5′-3′) | Product size (bp) |
| --- | --- | --- | --- |
| c96308_g2 | ttgcgctcccatcctcattt | taagaccagcccagagacca | 105 |
| c89788_g1 | cggaactgtttgcttcgctc | tgtggaccagttgacatgca | 141 |
| c90649_g3 | tacggcaaggtcaacaagca | ttccttgaagtgcggtccg | 135 |
| c95842_g3 | tgtttgcccagttctccgag | cacccttgaacccgtagtcc | 105 |
| c89769_g1 | actacccaccatgcccaaag | catcaacccactccccatcc | 135 |
| c75260_g1 | cttcgacgtgccctttggat | cctccacaacaagcagcaat | 101 |
| c88483_g1 | agagcattcttcaccggagc | gggaagaggaccgatcactg | 101 |
| 18S rRNA | cgttaacgaacgagacctca | cccagaacatctaagggcat | 144 |

Table S2 The differentially accumulated proteins between cold-stressed and unstressed jojoba leaves.

| **Protein ID^a^** | **Fold change (Means ± SD)** | **Coverage (%)** | **Number of peptides** | **Predicted subcellular location^b^** | **Homologous protein in Arabidopsis** | **Description in TAIR** | **Homologous protein in Swiss-Prot** | **Description in Swiss-Prot** |
| --- | --- | --- | --- | --- | --- | --- | --- | --- |
| **Photosynthesis** |  |  |  |  |  |  |  |  |
| c99581_g1\|m.23091 | 0.564±0.057 | 12.96 | 3 | C | AT4G14210 | PDE226, PDS, PDS3, PHYTOENE DESATURASE, PHYTOENE DESATURASE 3, PIGMENT DEFECTIVE 226 | P26294 | PDS3, PDS, PDE226 \| phytoene desaturase 3 |
| c83438_g1\|m.5086 | 0.501±0.081 | 23.48 | 4 | C | AT1G29930 | AB140, CAB1, CAB140, CHLOROPHYLL A/B BINDING PROTEIN 1, CHLOROPHYLL A/B PROTEIN 140, LHCB1.3, LIGHT-HARVESTING CHLOROPHYLL A/B-PROTEIN 1.3 | P12333 | CAB1, AB140, CAB140, LHCB1.3 \| chlorophyll A/B binding protein 1 |
| c106512_g6\|m.32699 | 1.663±0.041 | 12.58 | 2 | C | AT2G27510 | ATFD3, FD3, FERREDOXIN 3 | P27788 | ATFD3, FD3 \| ferredoxin 3 |
| c106174_g5\|m.22813 | 0.632±0.006 | 13.26 | 6 | C | AT1G68830 | STN7, STT7 HOMOLOG STN7 | Q9S713 | STN7 \| STT7 homolog STN7 |
| c74576_g1\|m.893 | 0.598±0.085 | 6.43 | 5 | C | AT3G14940 | PPC3, ATPPC3 \| phosphoenolpyruvate carboxylase 3 | P29196 | Phosphoenolpyruvate carboxylase 2 |
| c99596_g1\|m.12661 | 0.579±0.028 | 17.02 | 2 | C | AT3G26740 | CCL, CCR-LIKE | Q96500 | CCL \| CCR-like |
| c97454_g2\|m.8570 | 0.585±0.029 | 5.18 | 2 | C | AT5G18570 | ATOBGC, ATOBGL, CHLOROPLASTIC SAR1, CPSAR1, EMB269, EMB3138, EMBRYO DEFECTIVE 269, EMBRYO DEFECTIVE 3138, OBG A-2, OBG-LIKE PROTEIN | Q8L7L0 | EMB269, ATOBGC, CPSAR1 \| GTP1/OBG family protein |
| c84328_g1\|m.29249 | 0.641±0.028 | 13.29 | 4 | C | AT5G39830 | DEG PROTEASE 8, DEG8, DEGP8, DEGRADATION OF PERIPLASMIC PROTEINS 8 | Q9LU10 | Protease Do-like 8, chloroplastic |
| c100976_g2\|m.1101 | 0.625±0.003 | 35.06 | 6 | C | AT1G67090 | ribulose bisphosphate carboxylase small chain 1A | Q08183 | Ribulose bisphosphate carboxylase small chain 3, chloroplastic |
| c72428_g1\|m.7078 | 0.528±0.020 | 61.58 | 11 | C | AT1G67090 | RBCS1A \| ribulose bisphosphate carboxylase small chain 1A | Q08183 | Ribulose bisphosphate carboxylase small chain 3, chloroplastic |
| c115573_g1\|m.33947 | 0.655±0015 | 61.11 | 14 | C | AT5G38410 | RBCS3B \| Rubisco small subunit 3B | Q9LHP1 | Ribulose bisphosphate carboxylase (small chain) family protein |
| c87870_g1\|m.1028 | 0.505±0.006 | 24.95 | 8 | C | AT2G39730 | RCA, RUBISCO ACTIVASE | Q7X9A0 | RCA \| Ribulose bisphosphate carboxylase/oxygenase activase, chloroplastic |
| c96954_g1\|m.38356 | 0.581±0.018 | 18.83 | 2 | C | AT2G35370 | GDCH \| glycine decarboxylase complex H | O82179 | Glycine cleavage system H protein 2, mitochondrial |
| c73752_g2\|m.4483 | 2.009±0.708 | 11.23 | 2 | C | AT1G79530 | GAPCP-1 \| glyceraldehyde-3-phosphate dehydrogenase of plastid 1 | P04406 | Glyceraldehyde-3-phosphate dehydrogenase |
| c87458_g1\|m.25443 | 1.732±0.197 | 19.18 | 12 | C | AT1G79750 | ARABIDOPSIS THALIANA NADP-MALIC ENZYME 4, ATNADP-ME4, NADP-MALIC ENZYME 4, NADP-ME4 | Q9CA83 | ATNADP-ME4, NADP-ME4 \| NADP-malic enzyme 4 |
| c103546_g1\|m.23527 | 0.628±0.071 | 24.41 | 2 | C | AT3G62410 | CP12, CP12 DOMAIN-CONTAINING PROTEIN 1, CP12 DOMAIN-CONTAINING PROTEIN 2, CP12-2 | Q9LZP9 | CP12-2, CP12 \| CP12 domain-containing protein 2 |
| **Protein synthesis, folding and degradation** |  |  |  |  |  |  |  |  |
| c90649_g3\|m.25784 | 0.573±0.071 | 18.85 | 4 | Cy | AT2G44120 | Ribosomal protein L30/L7 family protein | P60039 | 60S ribosomal protein L7-3 |
| c29024_g1\|m.11194 | 2.252±0.139 | 13.35 | 6 | Cy | AT3G13580 | Ribosomal protein L30/L7 family protein | Q9LHP1 | 60S ribosomal protein L7-4 |
| c68355_g2\|m.34206 | 0.597±0.038 | 30.88 | 7 | Cy | AT1G08360 | Ribosomal protein L1p/L10e family | B7F845 | 60S ribosomal protein L10a-1 |
| c69884_g1\|m.29985 | 1.604±0.130 | 9.22 | 2 | Cy | AT3G24830 | Ribosomal protein L13 family protein | Q9LRX8 | 60S ribosomal protein L13a-2 |
| c141309_g1\|m.33212 | 0.629±0.052 | 32.11 | 2 | Cy | AT5G24510 | 60S acidic ribosomal protein family | P52855 | 60S acidic ribosomal protein family |
| c95991_g1\|m.10525 | 0.537±0.023 | 11.16 | 2 | Cy | AT5G37475 | Translation initiation factor eIF3 subunit | Q6INR1 | Translation initiation factor eIF3 subunit |
| c62232_g2\|m.35209 | 0.581±0.20 | 44.19 | 5 | Cy | AT4G38740 | ROC1 \| rotamase CYP 1 | Q39613 | Peptidyl-prolyl cis-trans isomerase |
| c76236_g2\|m.9241 | 0.460±0.037 | 11.45 | 7 | Cy | AT5G56030 | HSP81-2,HSP90.2,AtHsp90.2,ERD8,HSP81.2 | Q6INR1 | Hsp81.4, AtHsp90.4 \| HEAT SHOCK PROTEIN 81.4 |
| c99242_g3\|m.31903 | 1.504±0.038 | 13.33 | 3 | ER | AT1G52600 | Peptidase S24/S26A/S26B/S26C family protein | Q5RC30 | Signal peptidase complex catalytic subunit SEC11C |
| c102373_g1\|m.2301 | 1.510±0.043 | 5.11 | 5 | C | AT5G19690 | STAUROSPORIN AND TEMPERATURE SENSITIVE 3-LIKE A, STT3A | Q93ZY3 | STT3A \| staurosporin and temperature sensitive 3-like A |
| c150116_g1\|m.2862 | 0.614±0.039 | 13.63 | 11 | Cy, N, M | AT5G56030 | HSP81-2,HSP90.2,AtHsp90.2,ERD8,HSP81.2 | P36181 | Heat shock cognate protein 80 |
| **Defense and stress response** |  |  |  |  |  |  |  |  |
| c94453_g1\|m.22227 | 1.632±0.143 | 43.28 | 13 | Cy, N | AT1G20440 | COR47, RD17, AtCOR47 \| cold-regulated 47 | P31168 | Dehydrin COR47 |
| c94418_g1\|m.15081 | 0.634±0.016 | 41.18 | 4 | Cy, ER | AT3G12490 | ATCYSB,ATCYS6,CYSB \| ARABIDOPSIS THALIANA PHYTOCYSTATIN 6,cystatin B | Q06445 | Cysteine proteinase inhibitor |
| c68072_g1\|m.17149 | 0.540±0.012 | 37.12 | 10 | ER, S | AT1G17860 | ARABIDOPSIS THALIANA KUNITZ TRYPSIN INHIBITOR 5, ATKTI5 | P13087 | Miraculin |
| c79797_g1\|m.18714 | 0.582±0.035 | 49.68 | 6 | Cy | AT1G70850 | MLP34 \| MLP-like protein 34 | P85524 | Kirola |
| c94618_g1\|m.38026 | 0.628±0.054 | 20.82 | 4 | S | AT4G14630 | Germin-like protein 9 | Q9FLT3 | Germin-like protein subfamily 3 member 4 |
| c92573_g1\|m.34572 | 0.439±0.033 | 6.22 | 2 | C | AT3G11340 | UDP-DEPENDENT GLYCOSYLTRANSFERASE 76B1, UGT76B1 | U3UA11 | UDP-glucose iridoid glucosyltransferase |
| c93838_g1\|m.28470 | 0.562±0.030 | 35.80 | 9 | V, S | AT3G22060 | CRK25, CYSTEINE-RICH RLK (RECEPTOR-LIKE PROTEIN KINASE) 25 | Q9LRJ9 | Cysteine-rich repeat secretory protein 38 |
| c87374_g1\|m.9535 | 0.652±0.004 | 16.53 | 2 | Cy, CM | AT3G17020 | Adenine nucleotide alpha hydrolases-like superfamily protein | Q8VYN9 | Universal stress protein PHOS32 |
| **Vesicle transport** |  |  |  |  |  |  |  |  |
| c75260_g1\|m.21605 | 1.773±0.171 | 6.36 | 2 | G, V | AT5G22360 | ATVAMP714, VAMP714, VESICLE-ASSOCIATED MEMBRANE PROTEIN 714 | Q9FMR5 | ATVAMP714, VAMP714 \| vesicle-associated membrane protein 714 |
| c87179_g1\|m.23163 | 0.668±0.009 | 14.90 | 3 | G, C | AT5G53530 | VPS26A \| vacuolar protein sorting 26A | Q9FJD0 | Vacuolar protein sorting-associated protein 26A |
| c67735_g1\|m.35839 | 0.521±0.026 | 4.53 | 4 | G | AT2G14740 | ATVSR3, BINDING PROTEIN OF 80 KDA 2;2, BP80-2;2, UXS2, VACULOLAR SORTING RECEPTOR 3, VACUOLAR SORTING RECEPTOR 2;2, VACUOLAR SORTING RECEPTOR 3, VSR2;2, VSR3 | P93484 | Vacuolar-sorting receptor 1 |
| c87990_g2\|m.13700 | 0.658±0.056 | 33.02 | 7 | Cy, G | AT3G53610 | ATRAB8, ATRAB8B, ATRABE1A, RAB GTPASE HOMOLOG 8, RAB8 | O24466 | Ras-related protein RABE1a |
| c95275_g1\|m.15107 | 0.594±0.087 | 31.09 | 5 | Cy, G | AT5G17060 | ADP-RIBOSYLATION FACTOR B1B, ARFB1B, ATARFB1B | P91924 | ADP-ribosylation factor |
| c2874_g1\|m.27072 | 1.549±0.010 | 41.85 | 5 | Cy | AT3G49870 | ARLA1C, ATARLA1C \| ADP-ribosylation factor-like A1C | Q8VY57 | ADP-ribosylation factor-like protein 8a |
| c95135_g1\|m.4240 | 0.621±0.039 | 12.80 | 2 | ER | AT1G21900 | P24 SUBFAMILY DELTA 5, P24DELTA5 | Q9LQY3 | Transmembrane emp24 domain-containing protein p24delta9 |
| c55372_g1\|m.12728 | 0.551±0.034 | 11.98 | 3 | ER | AT1G21900 | P24 SUBFAMILY DELTA 5, P24DELTA5 | Q6IDL4 | Transmembrane emp24 domain-containing protein p24delta3 |
| **Lipid metabolism and transport** |  |  |  |  |  |  |  |  |
| c67023_g1\|m.21158 | 1.626±0.057 | 19.52 | 3 | Cy, M | AT2G44620 | MTACP1, MTACP-1 \| mitochondrial acyl carrier protein 1 | P53665 | Acyl carrier protein 1, mitochondrial |
| c65482_g1\|m.32535 | 1.513±0.105 | 12.52 | 8 | C | AT4G33510 | DAHP2, AtDAHP2, DHS2 \| 3-deoxy-d-arabino-heptulosonate 7-phosphate synthase, 3-DEOXY-D-ARABINO-HEPTULOSONATE-7-PHOSPHATE 2 | P27608 | Phospho-2-dehydro-3-deoxyheptonate aldolase 1, chloroplastic |
| c101515_g1\|m.24851 | 1.559±0.058 | 19.30 | 11 | C | AT1G54570 | PES1 \| phytyl ester synthase 1 | Q9ZVN2 | Acyltransferase-like protein At1g54570, chloroplastic |
| c99161_g4\|m.11080 | 0.485±0.016 | 7.67 | 2 | C | AT3G14225 | GLIP4 \| GDSL-motif lipase 4 | Q9LJP1 | GDSL esterase/lipase 4 |
| c98424_g1\|m.33351 | 0.509±0.039 | 21.60 | 2 | C, Cy | AT2G16005 | INTERACTOR OF SYNAPTOTAGMIN1, ROSY1 | Q54SW1 | Putative phosphatidylglycerol/phosphatidylinositol transfer protein DDB_G0282179 |
| c93174_g2\|m.33911 | 0.637±0.021 | 45.45 | 4 | C, ER | AT5G53560 | ATB5-A, CB5-E, ATCB5-E, B5 #2 \| ARABIDOPSIS CYTOCHROME B5 ISOFORM E, cytochrome B5 isoform E | P40934 | Cytochrome b5 |
| c32260_g1\|m.16818 | 0.533±0.018 | 43.70 | 4 | C, ER | AT2G32720 | ATCB5-B, CB5-B, CYTB5-D \| cytochrome B5 isoform B, ARABIDOPSIS CYTOCHROME B5 ISOFORM B | P49098 | Cytochrome b5 |
| c99948_g1\|m.3833 | 0.556±0.075 | 29.85 | 3 | C, ER | AT2G32720 | B5 #4, ATCB5-B, CB5-B, CYTB5-D \| cytochrome B5 isoform B, ARABIDOPSIS CYTOCHROME B5 ISOFORM B | P49098 | Cytochrome b5 |
| c98315_g1\|m.6595 | 1.502±0.052 | 20.98 | 4 | N | AT1G55260 | LTPG6 \| glycosylphosphatidylinositol-anchored lipid protein transfer 6 | O64864 | Protein YLS3 |
| c83245_g1\|m.26913 | 0.505±0.050 | 10.76 | 2 | C | AT3G57280 | FAX1 \| fatty acid export 1 | Q9ZVH7 | Protein FATTY ACID EXPORT 3, chloroplastic |
| **Signal transduction** |  |  |  |  |  |  |  |  |
| c106711_g4\|m.21306 | 0.616±0.016 | 20.83 | 2 | Cy, N | AT2G44310 | Calcium-binding EF-hand family protein | Q86JE1 | Recoverin family protein DDB_G0272130 |
| c95275_g1\|m.15106 | 0.64±0.087 | 31.09 | 5 | PM, C | AT2G38750 | ANNAT4, ANNEXIN 4, ATANN4 | Q9ZVJ6 | Annexin D4 |
| c100699_g1\|m.29246 | 0.569±0.032 | 23.12 | 3 | C | AT2G23070 | CKA4, cpCK2 \| CK2 alpha cp | B6F107 | Casein kinase II subunit alpha-2 |
| c101071_g1\|m.38683 | 0.648±0.044 | 8.33 | 2 | N | AT5G60550 | GRIK2, ATSNAK1 \| geminivirus rep interacting kinase 2 | Q5HZ38 | Serine/threonine-protein kinase GRIK2 |
| c89769_g1\|m.4220 | 0.626±0.018 | 7.76 | 3 | Cy | AT1G05010 | EFE, ACO4, EAT1 \| ethylene-forming enzyme | P31237 | 1-aminocyclopropane-1-carboxylate oxidase |
| c104002_g3\|m.3345 | 1.542±0.021 | 12.14 | 5 | S | AT2G17230 | EXL5 \| EXORDIUM like 5 | Q9SII5 | Protein EXORDIUM-like 5 |
| **ROS scavenging** |  |  |  |  |  |  |  |  |
| c89788_g1\|m.27963 | 1.742±0.079 | 48.68 | 5 | Cy | AT1G08830 | CSD1, AtSOD1, SOD1 \| superoxide dismutase 1, copper/zinc superoxide dismutase 1 | P93258 | Superoxide dismutase [Cu-Zn] 1 |
| c87338_g1\|m.26819 | 0.583±0.029 | 21.12 | 6 | C | AT1G32220 | NAD(P)-binding Rossmann-fold superfamily protein | Q9FVR6 | Uncharacterized protein At1g32220, chloroplastic |
| c84109_g1\|m.11754 | 0.644±0.004 | 60.14 | 11 | Cy | AT3G25530 | AtGLYR1, GLYR1, ATGHBDH, GHBDH, GR1 \| GLYOXYLATE REDUCTASE 1, glyoxylate reductase 1 | Q9LSV0 | Glyoxylate/succinic semialdehyde reductase 1 |
| c100983_g1\|m.4488 | 0.650±0.037 | 24.18 | 8 | C | AT1G67280 | ATGLYI6, GLYOXALASEI 6 | Q8W593 | Probable lactoylglutathione lyase, chloroplastic |
| c48364_g1\|m.16051 | 0.653±0.033 | 11.61 | 2 | Cy | AT3G09270 | ATGSTU8, GLUTATHIONE S-TRANSFERASE TAU 8, GSTU8 | P32110 | Probable glutathione S-transferase |
| c75533_g1\|m.30774 | 0.606±0.045 | 13.02 | 2 | C | AT2G47730 | ARABIDOPSIS THALIANA GLUTATHIONE S-TRANSFERASE PHI 8, ATGSTF5, ATGSTF8, GLUTATHIONE S-TRANSFERASE (CLASS PHI) 5, GLUTATHIONE S-TRANSFERASE PHI 8, GST6, GSTF8 | P46423 | Glutathione S-transferase |
| **Cell wall** |  |  |  |  |  |  |  |  |
| c102300_g3\|m.18078 | 0.649±0.063 | 27.51 | 8 | Cy, S | AT1G08200 | AXS2, UDP-D-APIOSE/UDP-D-XYLOSE SYNTHASE 2 | Q9SGE0 | UDP-D-apiose/UDP-D-xylose synthase 2 |
| c85273_g2\|m.38754 | 0.539±0.024 | 6.55 | 2 | S | AT4G34480 | O-Glycosyl hydrolases family 17 protein | Q94G86 | Glucan endo-1,3-beta-D-glucosidase |
| c84568_g3\|m.2339 | 1.547±0.146 | 5.68 | 4 | Cy | AT1G78570 | RHM1, ROL1, ATRHM1 \| rhamnose biosynthesis 1 | Q9SYM5 | Trifunctional UDP-glucose 4,6-dehydratase/UDP-4-keto-6-deoxy-D-glucose 3,5-epimerase/UDP-4-keto-L-rhamnose-reductase RHM1 |
| c103293_g11\|m.13685 | 0.660±0.054 | 20.00 | 5 | S | AT2G33590 | ATCRL1, CCR(CINNAMOYL COA:NADP OXIDOREDUCTASE)-LIKE 1, CRL1 | Q9SAH9 | Cinnamoyl-CoA reductase 2 |
| c98040_g1\|m.2444 | 0.621±0.057 | 14.29 | 2 | Cy | AT4G26220 | S-adenosyl-L-methionine-dependent methyltransferases superfamily protein | Q9C5D7 | Probable caffeoyl-CoA O-methyltransferase At4g26220 |
| c104594_g9\|m.6973 | 1.539±0.039 | 50.47 | 13 | S | AT5G05340 | PRX52 \| peroxidase 52 | Q9FLC0 | Peroxidase 52 |
| c83965_g1\|m.36104 | 1.614±0.138 | 5.73 | 2 | G | AT3G49720 | CGR2 | Q9M2Y6 | Probable pectin methylesterase CGR2 |
| **Cytoskeleton** |  |  |  |  |  |  |  |  |
| c101531_g3\|m.21226 | 0.604±0.017 | 50.11 | 18 | Cy | AT1G04820 | TUA4,TOR2 \| TORTIFOLIA 2,tubulin alpha-4 chain | Q9ZRR5 | Tubulin alpha-3 chain |
| c101531_g2\|m.17244 | 0.589±0.056 | 49.89 | 18 | Cy | AT1G04820 | TUA4,TOR2 \| TORTIFOLIA 2,tubulin alpha-4 chain | Q6VAF9 | Tubulin alpha-4 chain |
| c101416_g1\|m.39195 | 1.581±0.033 | 20.44 | 8 | Cy | AT1G04820 | TUA4,TOR2 \| TORTIFOLIA 2,tubulin alpha-4 chain | P06603 | Tubulin alpha-1 chain |
| c55616_g1\|m.11843 | 0.491±0.009 | 17.45 | 5 | Cy | AT5G23860 | TUB8 \| tubulin beta 8 | P29516 | Tubulin beta-8 chain |
| c87517_g3\|m.6212 | 0.614±0.026 | 26.52 | 7 | Cy | AT2G37620 | ACT1,AAc1 \| ARABIDOPSIS ACTIN 1,actin 1 | O81221 | Actin |
| **Transmembrane transport** |  |  |  |  |  |  |  |  |
| c96308_g2\|m.40831 | 1.549±0.067 | 5.64 | 5 | C, M, V | AT1G15690 | ARABIDOPSIS THALIANA V-PPASE 3, ATAVP1, ATAVP3, ATVHP1;1, AVP-3, AVP1, FUGU 5, FUGU5, VHP1 | P21616 | Pyrophosphate-energized vacuolar membrane proton pump |
| c93447_g1\|m.19008 | 0.562±0.008 | 15.55 | 3 | CM | AT4G35100 | PIP2;7, PIP3, PIP3A, PLASMA MEMBRANE INTRINSIC PROTEIN 2;7, PLASMA MEMBRANE INTRINSIC PROTEIN 3, PLASMA MEMBRANE INTRINSIC PROTEIN 3A, SIMIP | P93004 | Aquaporin PIP2-7 |
| c74667_g1\|m.15615 | 0.642±0.040 | 10.90 | 8 | V | AT4G30190 | AHA2, ATHA2, H(+)-ATPASE 2, HA2, PLASMA MEMBRANE PROTON ATPASE 2, PMA2 | Q03194 | Plasma membrane ATPase 4 |
| **RNA processing** |  |  |  |  |  |  |  |  |
| c104579_g4\|m.9638 | 1.506±0.049 | 8.97 | 3 | C | AT1G70200 | HIGH PHOTOSYNTHETIC EFFICIENCY 1, HPE1, RBD1 | – | – |
| c98803_g1\|m.6884 | 0.617±0.032 | 11.50 | 2 | N, Cy | AT1G51510 | Y14 | F4I9J7 | Y14 \| RNA-binding protein Y14 |
| c105802_g6\|m.38261 | 1.510±0.129 | 5.46 | 3 | C, M | AT1G12770 | EMB1586, EMBRYO DEFECTIVE 1586, INCREASED SIZE EXCLUSION LIMIT 1, ISE1 | Q8W4E1 | ISE1, EMB1586 \| P-loop containing nucleoside triphosphate hydrolases superfamily protein |
| c104085_g1\|m.38884 | 1.577±0.039 | 12.84 | 8 | N | AT3G18610 | ATNUC-L2, NUC-L2, NUC2, NUCLEOLIN 2, NUCLEOLIN LIKE 2, PARALLEL1-LIKE 1, PARLL1 | Q7XTT4 | PARLL1, ATNUC-L2, NUC-L2 \| nucleolin like 2 |
| **Carbohydrate metabolism** |  |  |  |  |  |  |  |  |
| c85559_g2\|m.39279 | 1.940±0.046 | 21.20 | 5 | C | AT1G32900 | GBSS1, GRANULE BOUND STARCH SYNTHASE 1 | Q43784 | Granule-bound starch synthase 1, chloroplastic/amyloplastic |
| c100924_g1\|m.1635 | 1.530±0.045 | 14.69 | 6 | C | AT4G39210 | APL3 \| Glucose-1-phosphate adenylyltransferase family protein | Q00081 | Glucose-1-phosphate adenylyltransferase large subunit 1 |
| c94446_g2\|m.14690 | 0.670±0.041 | 10.63 | 4 | Cy | AT1G06410 | ATTPSA, TPS7, ATTPS7 \| TREHALOSE -6-PHOSPHATASE SYNTHASE S7, trehalose-phosphatase/synthase 7 | Q9LMI0 | Probable alpha,alpha-trehalose-phosphate synthase [UDP-forming] 7 |
| **Secondary metabolism** |  |  |  |  |  |  |  |  |
| c95842_g3\|m.38113 | 1.829±0.065 | 5.21 | 3 | Cy | AT2G37040 | PAL1, ATPAL1 \| PHE ammonia lyase 1 | P45726 | Phenylalanine ammonia-lyase |
| c88483_g1\|m.14253 | 1.59±0.017 | 6.25 | 3 | Cy, N | AT5G08640 | FLS1 \| flavonol synthase 1 | Q9ZWQ9 | Flavonol synthase/flavanone 3-hydroxylase |
| **Miscellaneous and unknown proteins** |  |  |  |  |  |  |  |  |
| c98687_g3\|m.36150 | 0.599±0.059 | 19.87 | 4 | ER | AT4G17050 | UGLYAH, UREIDOGLYCINE AMINOHYDROLASE | Q8GXV5 | UGLYAH \| ureidoglycine aminohydrolase |
| c104846_g4\|m.5575 | 0.618±0.038 | 26.69 | 6 | – | AT4G15545 | PH-response transcription factor | Q93W28 | Uncharacterized protein At4g15545 |
| c86797_g2\|m.4370 | 1.56±0.063 | 13.92 | 2 | – | – | – | – | – |
| c66959_g1\|m.276 | 1.529±0.037 | 14.35 | 3 | – | – | – | – | – |
| c94059_g1\|m.29900 | 0.67±0.072 | 7.82 | 2 | Cy | – | – | Q8LBZ4 | OTU domain-containing protein At3g57810 |
| c99253_g1\|m.1462 | 0.667±0.088 | 6.13 | 2 | Cy | AT5G02100 | ORP3A, UNE18 \| OSBP(OXYSTEROL BINDING PROTEIN)-RELATED PROTEIN 3A,UNFERTILIZED EMBRYO SAC 18 | Q93Y40 | Oxysterol-binding protein-related protein 3C |
| c105933_g6\|m.10230 | 0.663±0.014 | 10.40 | 2 | C | – | – | Q0WWT7 | Rhodanese-like domain-containing protein 11, chloroplastic |
| c80310_g1\|m.23659 | 0.571±0.060 | 17.57 | 3 | C | – | – | Q94A65 | Rhodanese-like domain-containing protein 14, chloroplastic |
| c73601_g1\|m.15532 | 0.652±0.072 | 37.50 | 6 | – | AT3G16640 | AtTCTP2, TCTP \| translationally controlled tumor protein | P35681 | Translationally-controlled tumor protein homolog |
| c90750_g1\|m.16187 | 0.651±0.041 | 27.74 | 3 | – | – | – | – | – |
| c102036_g1\|m.5542 | 0.647±0.065 | 11.71 | 6 | M | ATMG01190 | ATP1 \| ATP synthase subunit 1 | P37211 | ATP synthase subunit alpha, mitochondrial |
| c96521_g1\|m.19368 | 0.637±0.025 | 9.39 | 3 | M | AT4G34030 | MCCB \| 3-methylcrotonyl-CoA carboxylase | Q9LDD8 | Methylcrotonoyl-CoA carboxylase beta chain, mitochondrial |
| c102946_g4\|m.4810 | 0.636±0.008 | 10.88 | 2 | – | – | – | Q556Z9 | Transmembrane protein DDB_G0273707/DDB_G0273361 |
| c103998_g2\|m.32249 | 0.630±0.036 | 32.43 | 4 | – | AT1G22380 | UGT85A3, AtUGT85A3 \| UDP-glucosyl transferase 85A3 | F8WKW1 | 7-deoxyloganetin glucosyltransferase |
| c103749_g3\|m.7839 | 0.610±0.070 | 20.11 | 2 | M | AT1G17530 | ATTIM23-1, TIM23-1 \| translocase of inner mitochondrial membrane 23 | Q9LNQ1 | Mitochondrial import inner membrane translocase subunit TIM23-1 |
| c96170_g1\|m.10154 | 0.602±0.076 | 12.67 | 2 | – | – | – | – | – |
| c19075_g1\|m.8582 | 0.591±0.040 | 20.00 | 8 | Cy | AT4G13940 | MEE58, SAHH1, EMB1395, HOG1, SAH1, ATSAHH1 \| EMBRYO DEFECTIVE 1395, HOMOLOGY-DEPENDENT GENE SILENCING 1, MATERNAL EFFECT EMBRYO ARREST 58, S-ADENOSYL-L-HOMOCYSTEIN HYDROLASE 1 | Q01781 | Adenosylhomocysteinase |
| c67672_g2\|m.23590 | 0.659±0.031 | 41.67 | 2 | – | – | – | P37219 | Abscisic stress-ripening protein 2 |
| c96043_g1\|m.7963 | 0.631±0.027 | 38.26 | 5 | – | – | – | Q53JF7 | Abscisic stress-ripening protein 5 |
| c94774_g1\|m.2494 | 1.558±0.013 | 37.50 | 8 | N | AT1G09200 | H3.1 \| histone 3.1 | Q76MV0 | Histone H3.2 |

^a^ The IDs in the protein database, all of them come from the customized protein database of jojoba. **^b^** The subcellular location information of DAPs came from that of homologous proteins in Arabidopsis in UniProt database, or TAIR (www.arabidopsis.org), if no such information was found in UniProt. C, chloroplast; N, nucleus; ER, endoplasmic reticulum; Cy, cytoplasmic matrix; M, mitochondria; CM, cytoplasmic membrane; S, apoplast and cell wall; G, golgi apparatus; V, vacuole.
